# The speed–curvature power law in *Drosophila* larval locomotion

**DOI:** 10.1098/rsbl.2016.0597

**Published:** 2016-10

**Authors:** Myrka Zago, Francesco Lacquaniti, Alex Gomez-Marin

**Affiliations:** 1 Laboratory of Neuromotor Physiology, IRCCS Santa Lucia Foundation, Via Ardeatina 306, 00179 Rome, Italy; 2 Department of Systems Medicine, Medical School, University of Rome Tor Vergata, Via Montpellier 1, 00133 Rome, Italy; 3 Centre of Space Biomedicine, University of Rome Tor Vergata, Via Montpellier 1, 00133 Rome, Italy; 4 Behavior of Organisms Laboratory, Instituto de Neurociencias CSIC-UMH, Av. Ramón y Cajal, Alacant, Spain

**Keywords:** power law, motor control, *Drosophila*, locomotion

## Abstract

We report the discovery that the locomotor trajectories of *Drosophila* larvae follow the power-law relationship between speed and curvature previously found in the movements of human and non-human primates. Using high-resolution behavioural tracking in controlled but naturalistic sensory environments, we tested the law in maggots tracing different trajectory types, from reaching-like movements to scribbles. For most but not all flies, we found that the law holds robustly, with an exponent close to three-quarters rather than to the usual two-thirds found in almost all human situations, suggesting dynamic effects adding on purely kinematic constraints. There are different hypotheses for the origin of the law in primates, one invoking cortical computations, another viscoelastic muscle properties coupled with central pattern generators. Our findings are consistent with the latter view and demonstrate that the law is possible in animals with nervous systems orders of magnitude simpler than in primates. Scaling laws might exist because natural selection favours processes that remain behaviourally efficient across a wide range of neural and body architectures in distantly related species.

## 1. Introduction

When we scribble our name on a piece of paper, the instantaneous angular speed is related to the local path curvature according to a power law [1]. The law is one of the best-studied characteristics of human voluntary movements, holding for hand drawing, pursuit eye movements, speech and walking [1–4]. Movements complying with the law are maximally smooth [2,4,5]. The law is not given *a priori*: even when the path is imposed, as in hand drawing, movement speed could, in principle, vary in infinite ways, as shown by systematic deviations from the power law for some movements [2,6]. Therefore, the law must result from physiological constraints, although its origin remains debated. According to one view, the law originates by decoding complex cortical processes; indeed, population vectors in motor cortex obey the power law during drawing [7]. According to another view, the law stems from simple harmonic motions [6]—such as those output by spinal central pattern generators (CPGs)—coupled with the viscoelastic properties of muscles [8].

To the best of our knowledge, the power law has only been studied in human and non-human primates [1–8]. Here, we report that *Drosophila melanogaster* larvae, whose movements are controlled by a much simpler neural system [9], display a speed–curvature power law while crawling. This demonstrates that the law can emerge from the interplay between relatively simple neural commands and biomechanics. Our findings support the view that, despite huge divergence in anatomy, functional complexity and ecological contingencies, basic principles of motor control resulting in efficient behaviour are shared across distantly related species [4,10].

## 2. Material and methods

Experimental procedures and tracking of larvae behaviour were the same as in [11]. Third-instar *Drosophila melanogaster* larvae in the foraging stage were washed in 15%-sucrose solution and transferred to a flat-lid arena coated with a 3%-agarose slab. Animals were tracked at 7 frames s^−1^, 90 μm pixel^−1^ for 5 min. Tracking was interrupted if the animal touched the plate boundaries. Custom-made tracking scripts [12] extracted the location of the centroid, head and tail from postural sequences. We used three groups of larvae exposed to different experimental conditions: overshoot, approach and dispersal [11,13]. Odour gradients were created by suspending a 10 μl droplet of ethyl butyrate from the arena top, out of animals’ reach.

For the main analyses, the *x, y* position samples of the centroid were low-pass filtered (second-order, zero-phase-lag Butterworth filter). A 0.07 Hz cut-off frequency was chosen after verifying that the power-spectrum density of the raw data was approximately flat up to ≈0.01 Hz, and then dropped rapidly. At 0.07 Hz, the power was down by ≈30 dB. We interpolated the filtered data with cubic splines, computed the time derivatives of the interpolating spline, instantaneous curvature *C*(*t*) and angular speed *A*(*t*) from standard differential geometry [1,3,14]. Least-squares orthogonal-regression of log_10_*A*(*t*) versus log_10_*C*(*t*) was performed to estimate the exponent (*β*) and the variance accounted for (*r*^2^) by the power law *A*(*t*) = *kC*(*t*)*^β^*. Statistically significant differences of *β* between experimental conditions were assessed using non-parametric tests (Kruskal–Wallis ANOVA by ranks followed by multiple comparisons), because the data were not normally distributed (Kolmogorov–Smirnov test).

## 3. Results

**Figure 1.**
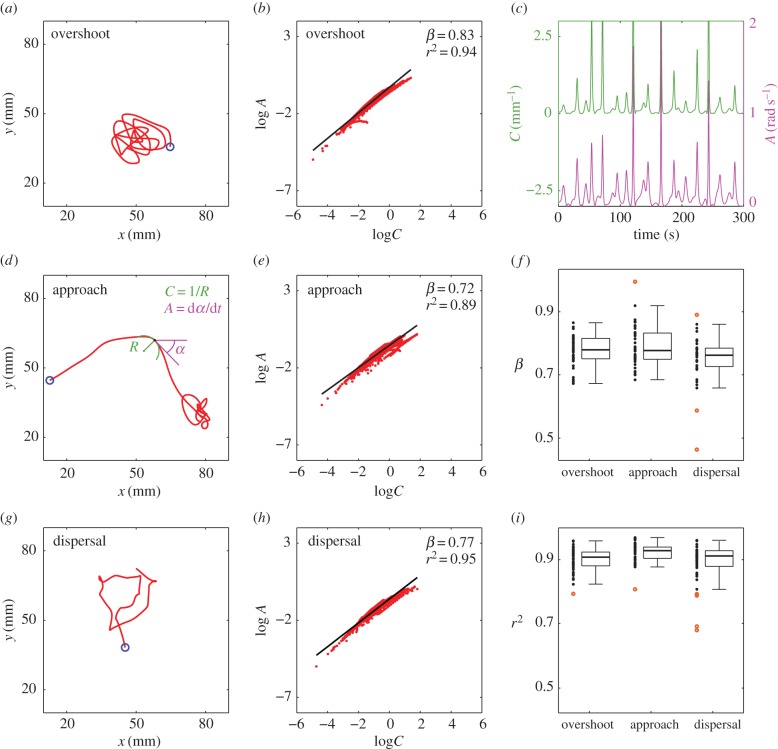
Relation between angular speed and path curvature in fly larvae tracing different trajectories. (*a*) Trajectory of the centroid position of one representative larva in the overshoot condition (blue circle indicates starting position). (*b*) Scatterplot of instantaneous angular speed *A* and local path curvature *C* on log_10_–log_10_ scale. All data points (red dots, *n* = 2100) sampled at equal time intervals along the same trajectory as in (*a*) were included. The data were fitted by the power function *A*(*t*) = *kC*(*t*)*^β^* (black line) with *β*-exponent and variance accounted for (*r*^2^) as indicated (top right). (*c*) Temporal evolution of the path curvature (green) and angular speed (magenta) for the same data as in (*a*–*b*). (*d*) Centroid trajectory of a larva in the approach condition. Key movement variables are identified at an arbitrary point along the trajectory: *C* is the curvature of the osculating circle of radius *R*, *α* is the phase angle of the tangent and the angular speed *A* is the time derivative of *α*. (*e*) Log–log plot of angular speed versus curvature for the same trajectory as in (*d*). (*f*) Summary boxplot statistics for *β*-exponent of individual animals in the three different groups: overshoot (*n* = 42), approach (*n* = 40) and dispersal (*n* = 41). Outliers are orange dots. (*g*) Centroid trajectory of a larva in the dispersal condition. (*h*) Log–log plot of angular speed versus curvature for the same trajectory as in *g*. (*i*) Summary boxplot statistics for *r*^2^ in the three groups. (Online version in colour.)

To induce animals to naturally ‘draw’ different types of trajectories, we tested different sensory environments [11]. In the overshoot condition during chemotaxis close to an odour source, the larval centroid traced complex trajectories resembling human scribbles (figure 1*a*). Trajectories were not associated with a constant progression speed or any simple kinematic pattern. Both the angular speed and path curvature were widely modulated, yet they covaried throughout (figure 1*c*). A log–log plot of angular speed versus curvature revealed a power law as a straight line whose slope corresponds to the power exponent (figure 1*b*). Power-law scaling extended over three or more orders of magnitude along both axes, consistent with typical requirements for robust power laws [15]. Similar results were obtained for all individual larvae in this condition.

Next, we tested larvae subjected to other sensory environments, resulting in different exploratory strategies and movement trajectories. In the approach condition, individuals reached an odour source at the opposite side of the arena via progressively more curved paths (figure 1*d*). In the dispersal condition, larvae searching for food in the absence of olfactory cues moved in arbitrary directions tracing highly variable paths (figure 1*g*). Overall, the power law did not depend on the type of exploratory movements: overshooting, approaching and dispersing larvae complied with the power law (figure 1*b*,*e*,*h*,*i*). The median value of the power exponent was 0.78 (interquartile range = 0.06, *n* = 42), 0.78 (interquartile range = 0.08, *n* = 40) and 0.76 (interquartile range = 0.06, *n* = 41) for the overshoot, approach and dispersal conditions, respectively (figure 1*f*). The distribution of the power exponents did not differ significantly between the three groups (Kruskal–Wallis *H*_2,123_ = 5.29, *p* = 0.071; multiple comparisons *p* > 0.05).

Similar results were observed for trajectories traced by the animal's hindmost part (tail): across all animals and conditions, the median *r*^2^ for the power law was 0.89 (interquartile range = 0.06, *n* = 123), and the median value of the power exponent was 0.74 (interquartile range = 0.09).

Moreover, the results were not affected substantially by using different frequency cut-offs in filtering the position data [16]. In the electronic supplementary material, figure S1 reports the results for the overshoot condition, but very similar results were obtained in the other conditions. The power law accounted well for the results, irrespective of filtering (median *r*^2^ > 0.85 over the tested range of frequency cut-offs, including no-filtering). The value of *β*-exponent varied with frequency cut-off, but only to a limited extent (median = 0.77, interquartile range = 0.06). A few individuals did not comply with the law (especially in the dispersal condition; see outliers as orange dots in figure 1*f*,*i*), confirming that it is not an obligatory outcome of our analyses.

## 4. Discussion

We have reported that a fundamental law of human control is at work in the humble maggot. The power law for voluntary movements in human and non-human primates may well have different origins [7] from those in crawling larvae. Yet, it is remarkable that the law is compatible with comparatively simple nervous systems, and that it holds for movements differing in speed by several orders of magnitude, such as those generated by humans and fly larvae. The non-trivial nature of the law is demonstrated by both theoretical considerations [5] and the empirical finding of violations in humans [2,6] and in some larvae here.

The power exponent for human hand drawing is generally close to 0.66 (so-called two-thirds power law [1]), but it becomes 0.73 when drawing in water [17], the latter value being close to the present values in larvae. Therefore, not only do we find in the larvae the geometric–kinematic constraint dictated by the power law, but also hints of dynamic constraints in the power exponent as recently found in humans [2,5,8,17], where its specific value depends on the viscosity of the medium (air or water for hand drawing, agar support and thin liquid coat for larval locomotion) and the trajectory shape.

In *Drosophila* larvae, multiple CPGs in the abdominal and thoracic segments of the nervous system generate peristaltic waves of muscle contractions along the body axis that enable crawling [9]. Curvature and crawling speed are regulated by two distinct processes. The degree of symmetry and synchrony of neural activity on each side of the nervous system controls the instantaneous direction of movement and therefore path curvature, straighter trajectories resulting from more symmetrical contractions in amplitude and timing [18], whereas frequency determines movement speed. It is then possible that the speed–curvature power law emerges from these patterns of neural activity transformed in oscillatory body motion, although suprasegmental nervous structures as well as sensory feedback also contribute to the net motor output [9]. It is also conceivable that the speed–curvature power law is solely due to viscoelastic muscle properties (as suggested by previous modelling studies [8]), although the presence of violations would argue against this interpretation. Future studies might use appropriate mutants to genetically disrupt the power law and provide generative mechanistic models [15] to elucidate the relative role of neural structures, body mechanics and sensory feedback control in the generation of such movement trajectories.

Typical strategies of environment exploration for available resources involve the execution of quasi-random walks with relatively straight stretches of locomotion alternating with abrupt changes of direction [9,11–14,18]. Scaling laws hold both at the whole-trajectory level and at the scale of local and instantaneous movements. At a macroscopic level, the probabilities of locomotor stretch sizes and idling-durations obey power-law distributions [19]. At a mesoscopic level, speed and curvature are related by the power law described here. Why do these scaling laws exist and why are they important in flies, and other organisms? One hypothesis is that they both entail optimal behaviour: Lévy-like foraging in sparse environments [19] and instantaneous movement smoothness for speed–curvature power law [2,4,5]. Scaling laws tend to be ubiquitous in nature, possibly because natural selection favours processes that remain behaviourally efficient across wide ranges of size and structure in different contexts [4,10] and across phyla.

## Supplementary Material

Figure S1. Effect of low-pass filtering on the power law.

## Data accessibility

Data available from the Dryad digital repository: http://dx.doi.org/10.5061/dryad.37sb7.

## Code accessibility

Code available from the SourceForge repository: https://sourceforge.net/projects/sos-track/.
