# Supplementary material for: The speed–curvature power law in Drosophila larval locomotion
Source: Biol Lett. 2016 Oct;12(10):20160597. doi: 10.1098/rsbl.2016.0597 (PMC5095195; doi:10.1098/rsbl.2016.0597)
Supplement: Figure S1. Effect of low-pass filtering on the power law. [file rsbl20160597supp1.docx]

*Electronic Supplementary Material for*

**The speed-curvature power law in Drosophila larval locomotion**

Myrka Zago, Francesco Lacquaniti and Alex Gomez-Marin

**
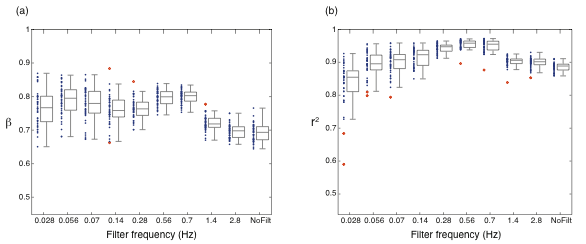
**

**Figure S1.** Effect of low-pass filtering on the power-law. The power function A(t)=kC(t)^β^ was fitted to the results of the larvae in the overshoot condition, after filtering the raw x, y position samples of the centroid at the frequency cutoff indicated on the abscissae. *Nofilt* denotes no filter whatsoever. Summary boxplot statistics are plotted for the β-exponent (*a*) and r^2^ (*b*). Outliers are orange dots.
